# Inducible viral receptor, A possible concept to induce viral protection in primitive immune animals

**DOI:** 10.1186/1743-422X-8-326

**Published:** 2011-06-28

**Authors:** Tirasak Pasharawipas

**Affiliations:** 1 Microbiology Unit, Department of Medical Science, Faculty of Science, Rangsit University, Pahonyothin Rd., Pathumthani, Thailand

**Keywords:** Pseudolysogen (PL), inducible viral receptor molecule, primitive-immune animals

## Abstract

A pseudolysogen (PL) is derived from the lysogenic *Vibrio harveyi *(VH) which is infected with the VHS1 (*Vibrio harveyi *Siphoviridae-like 1) bacteriophage. The lysogenic *Vibrio harveyi *undergoes an unequivalent division of the extra-chromosomal VHS1 phage genome and its VH host chromosome and produces a true lysogen (TL) and pseudolysogen (PL). The PL is tolerant to super-infection of VHS1, as is of the true lysogen (TL), but the PL does not contain the VHS1 phage genome while the TL does. However, the PL can become susceptible to VHS1 phage infection if the physiological state of the PL is changed. It is postulated that this is due to a phage receptor molecule which can be inducible to an on-and-off regulation influence by an alternating condition of the bacterial host cell. This characteristic of the PL leads to speculate that this phenomenon can also occur in high organisms with low immunity such as shrimp. This article proposes a hypothesis that the viral receptor molecule on the target cell can play a crucial role in which the invertebrate aquaculture animals can become tolerant to viral infection. A possible mechanism may be that the target cell disrupts the viral receptor molecule to prevent super infection. This concept can explain a mechanism for the prevention of viral infection in invertebrate animals which do not have acquired immunity in response to pathogens. It can guide us to develop a mechanism of immunity to viral infection in low-evolved-immune animals. Also, it can be an additional mechanism that exists in high immune organism, as in human for the prevention of viral infection

## Introduction

Lysogenic infection of the phage-bacterium relationship is considered similar to persistent infection. According to the lambda phage, its life cycle of lytic and lysogenic infection is dependent mainly on the role of the cI and Cro proteins. The predomination of cI protein maintains the phage in lysogenic stage while the Cro protein dominates the lytic infection [1,2]. It is claimed that the presence of cI protein in the lysogenic stage prevents super-infection by other lambda phage. However, if there is any stress such as starvation, the lambda phage in the lysogenic stage prefers transformation to the lytic pathway and causes the host cell death to release the phage particles [3]. Besides the lysogenic cell which might be called specifically as true lysogen (TL), there was a report of a pseudolysogen (PL) bacterial cell which is derived from a TL bacterium cell. The PL is named because of its property to prevent super-infection as does as TL. However, the PL does not contain the phage genome as does the TL. The PL can occur only in the case that the genome of temperate phage locates extra-chromosomally, plasmid-like genome, in the lysogenic cell. If the phage genome integrates into the bacterial chromosome, as most species of temperate phages do, the PL cannot be produced. The PL is reported in *Vibrio harveyi *which is infected with its specific temperate phage called VHS1 (*Vibrio harveyi *Siphoviridae-like1) [4]. It is explained that the PL is derived from an unequivalent division of the lysogenic chromosome and the phage genome. This causes one daughter cell containing the VHS1 phage genome and becomes the TL while the other one does not contain the VHS1 phage genome and subsequently is the PL.

It is well accepted that lysogen can tolerate (sometime called resistant, although the definition of tolerant and resistant is not exactly the same) a super-infection to the same phage. The explanation for this phenomenon has never been clearly explained. Besides, there are at least three different definitions of PL. The earliest report of PL concerns those infected cells that produce a defective phage genome as proposed by Cambell [5]. The other definition of PL is the phage that can produce spontaneously with a chromosomally integrated prophage [6]. In this article, the description of the PL is that it is derived from the unequivalent division of the lysogenic chromosome and the phage genome of the TL and can tolerate a specific phage infection without the existence of the phage genome [4,7]. This PL becomes sensitive to a VHS1 phage infection as result of a physical change such as a lowering growth condition for the PL [8]. The property of the PL that is described will be a guide line to explain the interference phenomenon and its relationship to the persistent viral infection in shrimp, cell culture and eventually human body.

## Interference phenomenon

Immunity to a second phage super-infection in lysogenic bacteria is similar to the interference phenomenon of the eukaryote; the target cell which is infected with the first virus would not be infected with the second viral infection. This can happen either among the same virus or the same subgroup (Homo-viral interference) [9,10]. It also occurs to the diverse genera (hetero-viral interference) [11-13]. There are different explanations for this phenomenon. The first explanation is that cellular enzymes which are required for the second viral infection are inhibited by the first virus which uses up the cellular resources and prevents the second virus to replicate [14]. In another explanation, the first virus generates defective interfering particles to compete for the replication resources to prohibit a second viral infection [15-17]. In a third report, the first viral infection induces the cell to synthesize protective substances to prevent super-infection of the virus. Some of these substances are virus specific, some are non-specific [18,19]. Interferon has been highly believed to play a role of interference [20]. However, there is also a contradictory report [21]. Thus, the mechanism of interferon to inhibit the viral infection has never been fully explained. According to these mentioned theories, there is no conclusive evidence about the mechanism of the interference phenomenon.

On the other hand, the interference phenomenon is not always true since dual viral infection in the same cell has been reported [22,23]. This means two different viruses can infect the same cell. Moreover, triple viral infection in the same culture cell is also presented [24]. It is reported that Dengue virus, Densovirus and Japanese encephalitis virus can infect in the same mosquito cell of c6/36 cell line. Obviously, these findings suggest that the interference phenomenon does not always exist as stated. This questions why some cells show interference to viral infection while c6/36 cells can be infected with multi-viral infections as studied by Kanthong et al [24]. The reason that interference phenomenon does not occur in this case might be that the three viruses enter the c6/36 cell by different receptor molecules. The receptor usage of the Japanese encephalitis virus and Dengue virus is reported to be distinct [25]. Unfortunately, the Densovirus receptor molecule has not been reported. More study of the other dual or multiple viral infections in the same cell together with the identification of the viral receptors are the interesting subjects to study to explain this interference phenomenon.

Hence, we present the hypothesis based on the inducible viral receptor concept that the host cell disrupts the expression of the receptor molecule after the first virus enters the cell. This mechanism can prevent not only the same virus entry (homo-viral interference) but also any other viruses (hetero-viral interference) that use the same viral receptor for entry into the target cell [26,27]. If this mechanism is influenced by interferon, further prove is needed. The phenomenon of the polyspermy block by the egg-sperm fertilization [28] may also be applied as a similar mechanism of the viral-cell interference phenomenon. However, it should be noted that prevention of polyspermy of an egg is to control the genetic inheritance of the parent. Prevention of poly-infection of viruses in the same target cell requires further explanation.

## Persistent viral infection in shrimp

In the mean time, there were independent observations in the shrimp aquaculture industry concerning the persistent infection of various viruses such as *Monodon baculovirus *[29], Yellow head virus [30,31], White spot virus [32,33] and Taura syndrome virus [34]. These viruses were isolated in shrimp during the first few years of a massive epidemic infection. The infected shrimp can be raised and harvested with normal size as in the uninfected shrimp [30,31]. However, the isolated viruses from these persistent viral infected shrimp cause the native shrimp to die [34]. Thus, the virulent existence of the viruses contradicts the explanation that shrimp resistance to the virus is due to viral mutation [34]. Additionally, it has been reported that these viral persistent shrimp are more sensitive to an inappropriate condition such as the high density of shrimp, the range of pH and oxygen amounts in the ponds [30,34]. Instead of stating that these persistent viral infected shrimp are resistant to the viruses, they should be called 'tolerant to the viruses' because the shrimp can exhibit tolerance only in optimal conditions [31,34]. In previous reports, it was claimed that the phenomenon of viral toleration in shrimp seems to be related to a specific mechanism. Thus, if the shrimp are tolerant to a specific virus, it would not tolerate other viral infections [30,34,35]. In addition, although shrimp do not have acquired immunity, there are some reports indicating that vaccination to some shrimp viruses is possible [33,36]. The mechanism to explain the vaccination for the immunity in shrimp was not explained in the report.

To explain the persistent viral infection in shrimp by the inducible receptor hypothesis; it can be explained that after viral infection into the cells of the target organ, the neighboring cells which are not infected process a mechanism to disrupt the viral receptor molecule so the virus cannot attach to them. This can prevent the virus to spread to the entire organ of shrimp to cause death. It should be mentioned that these persistent viral infected shrimp do not show any gross sign of pathogenesis of persistent viral infection and the virus can be detected by only the sensitive techniques such as the polymerase chain reaction (PCR) [34,37]. This means the amounts of the virus are very small and are limited in the persistent viral infected shrimp. However, if these persistent viral infected shrimp are raised in an inappropriate condition as mentioned above, they become infected and die more easily than the uninfected shrimp [31,34].

## Persistent viral infection in cell culture

In addition to using the viral inducible hypothesis to explain the appearance of PL in the VHS1-infected VH and the persistent viral infection in shrimp, the concept can be applied to interpret the report of Burivong et al [38] which concerns the cell culture of Densovirus infected C6/36 cell lines. The researchers reported that the sub-passage of the cell line for 9 passages decreases the Denzovirus persistent infection from 92% in the cell line to be about 20%. The researchers assumed that the decrease of the persistent cell line may be related to an interference infection of defective mutated virus [15-17]. However, the defective viruses have never been isolated or identified. With the report of the same group of researchers in a later report [39], the Densovirus persistently infected c6/36 cells resulted in less CPE (cytopathic effect) than the naïve c6/36 cells when they were super-challenged with the Dengue 2 virus [38,39]. If the result of the decrease of the Denzovirus persistent infection during the sub-passage is due to the influence of the defective interference (DI) as claimed by the researchers, Densovirus persistent cell should show similar levels of CPE with the naïve cell when both are super-challenged with the Dengue 2 virus because it should have no relationship between the existence of the DI particle of Densovirus and the Dengue 2 viral particle.

Based on the inducible viral receptor concept, the result of Burivong et al [38,39] could be simply explained that the Densovirus and Dengue 2 virus share the common receptor molecule to infect the c6/c36 cell [40-42]. It means that the cell which is persistently infected with the Densovirus down-regulates the common viral receptor molecule so the Dengue 2 virus can not penetrate into the Densovirus persistent cell as easier than the naïve c6/36 cell line in which the viral receptor molecules are intact and susceptible to viral attachment. Thus, the naïve cell line is more susceptible to the Dengue 2 infection than the Densovirus infected cell. However, Densovirus and Dengue virus are reported to simultaneously infect in the same cell [22-24]. The inducible viral receptor concept itself, at this moment, cannot explain and confirm the phenomenon that both interference and dual viral infection can occur in the same time. At present, the receptor molecule of Densovirus has not been identified although different researchers have reported that Dengue virus enters the target cell by various distinct types of molecules [43-46]. Accordingly, an assumption is that the Densovirus might also use more than one kind of receptor molecule as the Dengue virus does. At least one of those molecules is a common receptor molecule to allow both viruses to attach to the target cell. Thus, this can cause the interference phenomenon between the Densovirus and Dengue virus. In the mean time, the other receptor molecules are the specific receptor molecules for each virus to attach to the target cell. Although the Dengue virus and Densovirus share a common receptor molecule, both can use alternative molecules to enter the target cell if the dominant one is not present. So, both viruses can perform either interference phenomenon or dual infection. However, there is not any evidence to support this assumption.

## Persistent viral infection in human

In higher animals and humans who possess acquired immunity, there are some examples of viral infection which can be explained by the viral inducible receptor hypothesis. In case of persistent chronic hepatitis B carriers, the virus particles are produced continuously without any evidence of disease but cause pathological transmission to other humans [3,47]. However, there is the question why the virus does not infect the neighboring cell in the liver although the viral particle is still active and can horizontally transmit to other people. Accordingly, how do the neighboring cells prevent the wide spread viral infection. As mentioned previously, there are reports concerning the generation of the defective interfering particles which lack the necessary component(s) to generate productive infection [15-17]. This should be questioned because although it is true that defective interfering particles are produced, the complete viral particles should also be generated and cause infection or transmission as well. The appearance of defective viral particle might be just a co-incidence of viral interference. Accordingly, the 'viral inducible receptor' hypothesis can be applied to explain this question. The neighboring cells in the liver adapt to inhibit the molecule that plays a role of viral receptor. This makes the cell tolerant to homogenous infection of the Hepatitis B virus in the neighboring cell. This can also explain of the existence of chronic persistent Hepatitis B carriers who can be vulnerable (compared to normal individuals) to severe liver damage if they are exposed to the other liver causing pathological substances such as alcohol and other toxic substances [48]. However, with the viral inducible receptor hypothesis, it does not preclude the possibility that cytokine, such as interferon, might play this role to down regulate the neighboring cell and inhibit the viral infection. Although, Interferon was claimed to play the role of interference, the direct injection of interferon to prevent Hepatitis B virus has not been reported to be promising [48,49]. However, it is still possible that interferon plays an indirect role to regulate the viral receptor. More study is required.

## Conclusion

This paper presents an additional theory of an inducible viral receptor concept that the cell, in general, can adapt itself to prevent the viral infection. This process should be named as tolerant, instead of resistant, mechanism since the cell loses its prevention mechanism if exposed to inappropriate conditions such as low resources and toxic substance. This inducible viral receptor hypothesis can be used to explain the incidences by (1) the VH-PL phenomenon, (2) the toleration to viral infection in shrimp (3) the alternative interpretation of the reports of the Densovirus persistent C6/C36 cell and (4) the persistent infection of the Hepatitis B viral particle in the carriers. Mainly, it explains that each individual cell has an ability to learn to prevent the secondary viral infection by itself after the primary viral infection. This mechanism can explain the phenomenon of the viral toleration in either the low or high immune organism. In high immune organisms, the mechanism might be the additional pathway to help the memory lymphocytes to respond to the latter viral infection more promptly and effectively. In case of the primitive immune animals which lack lymphocyte to create the memory lymphocyte to respond to prevent the secondary viral infection, the phenomenon of the inducible viral receptor can explain the incidence of viral protection by the cell toleration as witness from the incidences in VH and shrimp. Moreover, it can explain why some researchers found that vaccination is possible in shrimp which possess lower immunity and do not have lymphocyte to create adaptive immune response and memory cells. However, this hypothesis still cannot explain it all especially the dual or multiple infection of the viruses in the same cell. I do wish more researchers would investigate the possibility of this concept.

## Abbreviation

PL: pseudolysogen; VH: *Vibrio harveyi*; VHS1: *Vibrio harveyi *siphoviridae-like 1 phage; TL: true lysogen.

## Competing interests

The author declares that he has no competing interests

## Authors' contributions

TP prepares and writes up the whole manuscript. He also read and approved the final manuscript.
